# Acute abdominal pain due to atypical bilateral adrenal infarction in acute myeloid leukemia with alterations related to myelodysplasia: A case report

**DOI:** 10.1002/ccr3.7925

**Published:** 2023-09-27

**Authors:** Risa Hirata, Masaki Tago, Shun Yamashita, Satsuki Yamamoto, Shizuka Yaita, Yuka Hirakawa, Maiko Ono, Shu‐ichi Yamashita

**Affiliations:** ^1^ Department of General Medicine Saga University Hospital Saga Japan; ^2^ Department of General Medicine Karatsu Municipal Hospital Karatsu Japan

**Keywords:** adrenal insufficiency, infarction/diagnostic imaging, adrenal glands, leukemia/myeloid/acute/case reports

## Abstract

**Key Clinical Message:**

Acute myeloid leukemia (AML) can cause acute abdomen following adrenal insufficiency or adrenal infarction. Therefore, when diffusely enlarged adrenal glands and adrenal insufficiency of unknown cause are seen in a patient presenting with acute abdomen, adrenal infarction due to AML, or other hematologic diseases should be ruled out.

**Abstract:**

A 49‐year‐old man developed acute abdominal pain following adrenal insufficiency and was diagnosed with acute myeloid leukemia (AML) with myelodysplasia‐related changes. Because AML can cause acute abdominal pain due to adrenal infarction following adrenal insufficiency, a patient with these conditions should be ruled out adrenal infarction due to AML or other hematologic diseases.

## Graphical abstract

Thoracoabdominal computed tomography with contrast enhancement on admission revealed diffusely enlarged bilateral adrenal glands with diffuse internal low‐attenuation areas. We report a case of abdominal pain and adrenal insufficiency due to AML‐induced adrenal infarction.

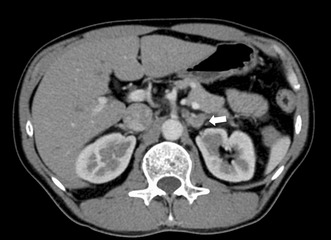

## 1 INTRODUCTION

The most common causes of abdominal pain are gastrointestinal diseases such as appendicitis, cholelithiasis, and intestinal obstruction; other common causes include urologic and gynecologic diseases. Furthermore, endocrinologic diseases such as ketoacidosis and adrenal insufficiency, systemic diseases such as vasculitis, and dermatologic diseases should be considered as differential diagnoses when the cause of acute abdomen cannot be identified.

The causes of adrenal insufficiency include autoimmune diseases; infectious diseases such as tuberculosis; metastases of malignancies such as lung cancer, breast cancer, stomach cancer, colon cancer, and lymphoma; adrenal hemorrhage; and drugs.^1^ The pathogenesis of adrenal insufficiency caused by acute leukemia involves infiltration of tumor cells, hemorrhage, infarction, and infection.^2^ Although hematologic diseases can cause adrenal infarction, acute myeloid leukemia (AML) rarely causes acute abdominal pain with adrenal infarction.^3^ In addition, in most cases of adrenal infarction, computed tomography (CT) images reveal diffuse enlargement of the adrenal glands.^4^, ^5^ Adrenal enlargement is mainly caused by adenoma, adrenal cortical hyperplasia, and metastatic infiltration.^6^ Less commonly, hematologic diseases such as unclassifiable myelodysplastic syndrome, myeloproliferative neoplasms,^7^ and myelodysplastic syndrome^8^ can cause adrenal infarction, which leads to adrenal enlargement.^5^

We herein report a case of acute abdominal pain and adrenal insufficiency due to AML‐induced adrenal infarction in a patient who presented with diffuse adrenal enlargement on CT imaging.

## 2 CASE PRESENTATION

### 2.1 Patient information

**FIGURE 1:**
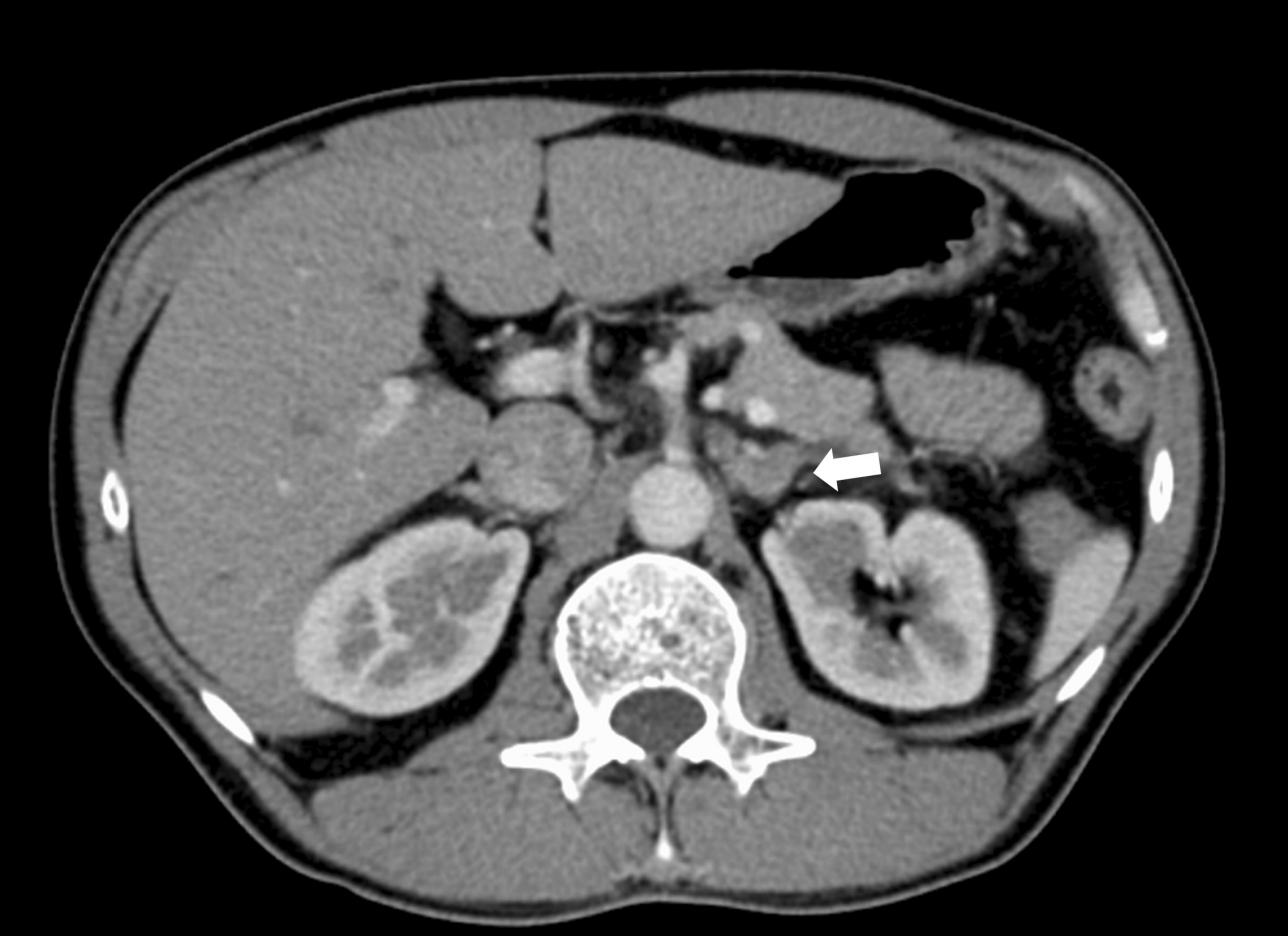
Thoracoabdominal computed tomography with contrast enhancement 16 days before the patient visited our hospital. Imaging revealed mild enlargement of the left adrenal gland and adipose tissue turbidity surrounding the left adrenal gland (arrow).

A 49‐year‐old healthy Japanese man without a history of regular health check‐ups or hospital visits presented with a 2‐month history of general fatigue and constipation. He felt discomfort in the left side of his abdomen while sleeping 16 days previously, and the discomfort gradually developed into pain and worsened. Although abdominal CT at another hospital revealed adipose tissue turbidity surrounding the left adrenal gland (Figure 1), the cause of the abdominal pain could not be detected, and the patient was discharged after spontaneous disappearance of the pain for a few days. On the day of admission to our hospital, he felt discomfort similar to that experienced 16 days previously in the right side of his back after lunch, and the discomfort gradually worsened into pain. He rated the intense pain in his right back, described as excruciating, as 9 to 10 on a 10‐point numeric rating scale. Analgesics were ineffective, and he was referred and admitted to our hospital for further examination. His systolic blood pressure had decreased by approximately 20 mmHg for the past 2 months and had ranged from 80 to 90 mmHg for the past 2 weeks. He had a smoking history with a Brinkman index of 580 and consumed more than 50 g/day of alcohol. The patient's family history included colorectal cancer in his father.

### 2.2 Clinical findings

**FIGURE 2:**
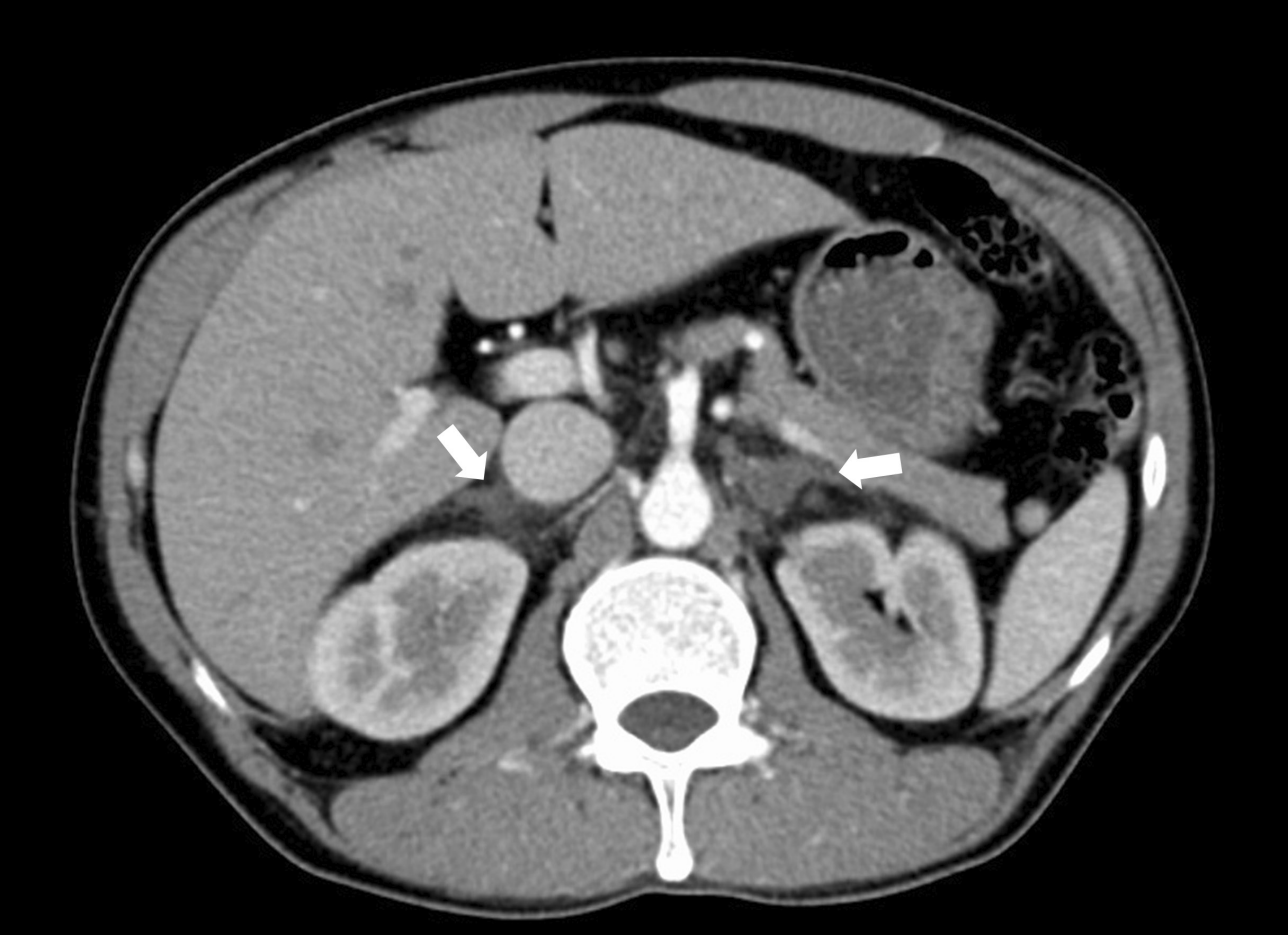
Thoracoabdominal computed tomography (CT) with contrast enhancement on admission. Imaging revealed diffusely enlarged bilateral adrenal glands with diffuse internal low‐attenuation areas and increased CT value in the surrounding adipose tissue (arrows).

**FIGURE 3:**
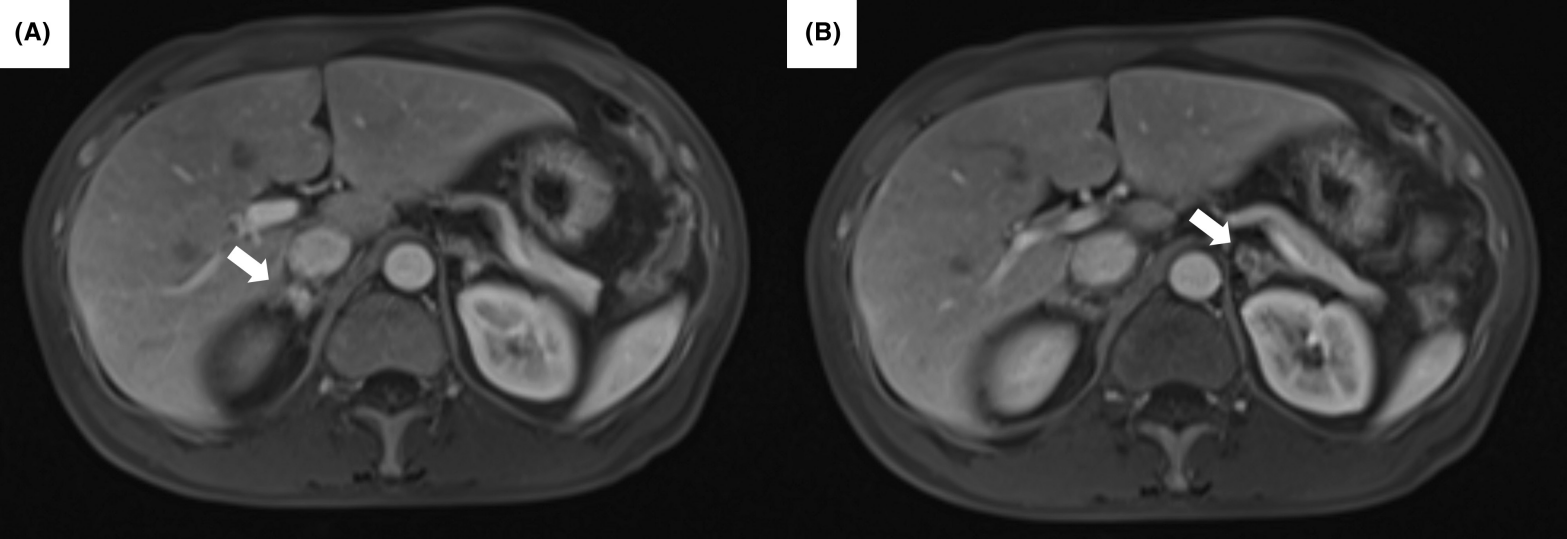
Abdominal magnetic resonance imaging with contrast enhancement. Magnetic resonance imaging showed that the adrenal glands were enlarged but smaller than on computed tomography on admission (A) and (B). Although post‐contrast T1‐weighted images revealed an area of partial low density, the contrast enhancement of the bilateral adrenal glands was good.

On admission, he was alert, and his body temperature was 38.5°C, heart rate 101 beats/min, blood pressure 148/84 mmHg, respiratory rate 18 breaths/min, and oxygen saturation 96% on room air. Physical examination revealed pallor of the eyelid conjunctiva, a soft and flat abdomen, and tenderness throughout the whole abdomen with the strongest point in the epigastrium and no signs of peritoneal irritation. His back pain was spontaneous only, with no significant findings on physical examination. The results of the blood tests are shown in Table 1. The blood tests showed leukopenia, anemia, and thrombocytosis. Mild abnormalities in the coagulation system were observed, while no abnormalities were found in the blood glucose or electrolyte levels. Thyroid‐stimulating hormone, free thyroxine, growth hormone, luteinizing hormone, follicle‐stimulating hormone, soluble interleukin 2 receptor, somatomedin C, and prolactin were all within the reference range. The antinuclear antibody level was normal, and the anticardiolipin antibody level was 2 U/mL. On the third day of admission, the adrenocorticotropic hormone level was 0.30 pmol/L, and the cortisol level was 775 nmol/L. An electrocardiogram exhibited sinus rhythm, and a chest radiograph revealed no abnormalities. Thoracoabdominal CT with contrast enhancement revealed diffusely enlarged bilateral adrenal glands with an increased CT value in the surrounding adipose tissue. There were no high‐attenuation areas suggesting hemorrhage within the adrenal glands, but low‐attenuation areas suggesting adrenal infarction were observed (Figure 2). CT revealed neither splenomegaly nor findings suggesting a cause of abdominal tenderness other than the enlarged adrenal glands. Additionally, considering the patient's history of hypotension and fatigue, we suspected adrenal insufficiency and began administration of hydrocortisone at 200 mg daily. We subsequently controlled his pain with fentanyl and started aspirin for platelet hyperplasia. Three days after admission, his platelet count decreased to a normal level of 289 × 10^9^/L, and the aspirin was stopped. The abdominal tenderness disappeared in 4 days. Abdominal magnetic resonance imaging with contrast enhancement on the third day of hospitalization showed that the adrenal glands were enlarged but smaller than on admission. Post‐contrast T1‐weighted images revealed an area of partial low density suggesting ischemia in the left adrenal gland; however, most of the blood flow was preserved (Figure 3).

**TABLE 1:** Blood tests on admission.

Parameter	Value	Reference range	Parameter	Value	Reference range
WBCs (×10^9^/L)	3.5	3.9–9.8	Uric acid (mmol/L)^a^	0.25	0.22–0.46
Neutrophils (%)	79.1	41.8–73.8	Amylase (nkat/L)	950	733–2200
Lymphocytes (%)	14.6	18.3–47.5	Creatine kinase (nkat/L)	1517	983–4133
Eosinophils (%)	0.0	0.0–5.6	Glucose (mmol/L)	13.2	4.1–6.1
RBCs (×10^12^/L)	2.78	4.10–5.30	Hemoglobin A1c (%)^a^	5.8	4.9–6.0
Hemoglobin (g/L)	100	135–176	Sodium (mmol/L)	141	138–146
Hematocrit (L/L)	0.293	0.407–0.501	Potassium (mmol/L)	3.3	3.6–4.9
Platelets (×10^9^/L)	900	86–123	Chlorine (mmol/L)	105	99–109
PT‐INR	1.14	0.9–1.10	Calcium (mmol/L)	2.17	2.20–2.52
APTT (s)	27.4	25.0–40.0	CRP (nmol/L)	60	0–13
Fib (g/L)	3.54	2.00–4.00	ACTH (pmol/L)^a^ ^,^ ^b^	0.30	1.6–13.9
D‐dimer (mg/L)	1.12	0.00–1.00	Cortisol (nmol/L)^a^ ^,^ ^b^	775	172–497
Total protein (g/L)	70	66–81	TSH (μIU/mL)	1.29	0.50–5.00
Albumin (μmol/L)	616.91	616.91–767.38	Free thyroxine (pmol/L)	12.87	11.58–21.88
AST (nkat/L)	333	217–500	GH (ng/mL)^a^	1.51	0.00–2.47
ALT (nkat/L)	483	167–700	LH (mIU/mL)^a^	6.45	2.20–8.40
γ‐GTP (nkat/L)	950	217–1067	FSH (mIU/mL)^a^	3.64	1.80–12.00
Total bilirubin (μmol/L)	5.13	6.84–25.66	Somatomedin C (ng/mL)^a^	83	88–246
LDH (nkat/L)	3850	2067–3700	Prolactin (ng/mL)^a^	2.85	4.29–13.69
ALP (nkat/L)	3983	1767–5367	Antinuclear antibody^a^	<40 times	<40 times
Blood urea nitrogen (mmol/L)	3.18	2.86–7.14	sIL2R (U/mL)^a^	379	121–613
Creatinine (μmol/L)	57.46	57.64–94.59	Anticardiolipin antibody (U/mL)^a^	2	<10

Abbreviations: ACTH, adrenocorticotropic hormone; ALP, alkaline phosphatase; ALT, alanine aminotransferase; APTT, activated partial thromboplastin time; AST, aspartate transaminase; CRP, C‐reactive protein; Fib, fibrinogen; FSH, follicle‐stimulating hormone; γ‐GTP, γ‐glutamyl transpeptidase; GH, growth hormone; LDH, lactate dehydrogenase; LH, luteinizing hormone; PT‐INR, prothrombin time–international normalized ratio; RBCs, red blood cells; sIL2R, soluble interleukin 2 receptor; TSH, thyroid‐stimulating hormone; WBCs, white blood cells.

^a^ Laboratory tests performed on the third day of hospitalization.

^b^ After administration of hydrocortisone.

### 2.3 Diagnostic assessment

**FIGURE 4:**
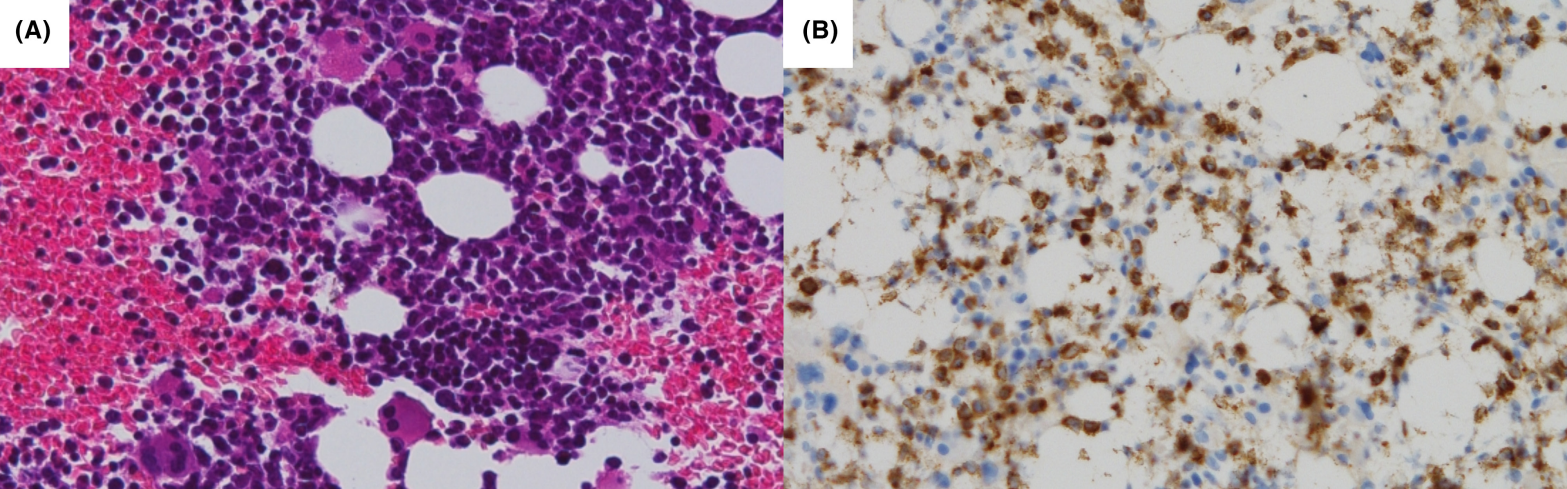
Histopathological findings of bone marrow. Histopathological examination showed a mixture of blood cells of three lineages, with the granulocyte lineage dominated by immature blasts (A). Approximately 50% of those cells were positive for myeloperoxidase staining (B).

The adrenocorticotropic hormone and cortisol levels before the administration of hydrocortisone on admission could not be measured because the patient visited outside regular hours. A rapid adrenocorticotropic hormone test showed a low cortisol response of 140 nmol/L before loading and 286 nmol/L after 60 min, suggesting primary adrenal insufficiency. Furthermore, a peripheral blood panel on admission showed an erythroblast level of 24.5%, megakaryocytes, promyelocytes, and myelocytes. A bone marrow puncture and biopsy were performed to rule out hematologic disease. Histopathological examination of the bone marrow revealed an atypical myeloblast level of 22.4% with positive myeloperoxidase staining (Figure 4). Background cells exhibited dysplastic features such as megakaryocytes with multiple nuclei, multinucleated erythroblasts, and multinucleated megakaryocytes. Pathological examination of the bone marrow revealed the notable presence of immature cells among granulocytes, a relative reduction in erythroblasts, and an increase in megakaryocytes. A pathological increase in iron granulocytes was not observed, and findings suggestive of erythroleukemia were absent; however, the observations remained consistent with myelodysplastic syndrome. Although the patient did not have chromosomal or genetic mutations, including t(8:21) translocation, the histopathological analysis showed remarkable myelodysplastic changes. Flow cytometry showed 5.9% blasts, negativity for CD56, and positivity for myeloperoxidase and CD177. Furthermore, WT1mRNA was positive.

### 2.4 Diagnosis

The patient was therefore diagnosed with AML with myelodysplasia‐related changes (French‐American‐British Classification M2).

### 2.5 Therapeutic interventions

Although there is no established treatment for AML with myelodysplasia‐related changes,^9^ standard therapy involving azacitidine at a dosage of 115 mg per administration for 7 consecutive days (115 mg/course) was promptly commenced on Day 10. Following admission, the patient received intravenous hydrocortisone at a dosage of 200 mg/day, resulting in improvement in the abdominal tenderness and hypotension. The dosage was then gradually reduced, and from Day 9, it was decreased to 50 mg/day.

### 2.6 Follow‐up, and outcome of interventions

Eventually, because of the improvement of adrenal function, hydrocortisone administration was discontinued on Day 26 at a final dosage of 30 mg/day. Abdominal contrast‐enhanced CT imaging performed on Day 35 revealed an improvement in the bilateral adrenal enlargement. The previously observed areas of poor contrast enhancement were no longer present, and the surrounding fat tissue turbidity had also improved. However, subsequent treatment for AML showed limited effectiveness. On Day 40, the standard treatment regimen involved the administration of idarubicin at a dose of 18 mg for 3 days and cytarabine at a dose of 150 mg for 7 days. On Day 161, allogeneic peripheral blood stem cell transplantation was performed, resulting in the achievement of remission. However, the patient died 20 months after the diagnosis.

## 3 DISCUSSION

The present report describes an uncommon case of acute abdomen following adrenal infarction caused by AML. Although adrenal diseases such as adrenal crisis and adrenal infarction can cause acute abdomen manifesting as acute back, chest, and upper abdominal pain,^4^, ^8^, ^10^, ^11^ such cases are rare in actual clinical practice. Previous studies regarding abdominal pain showed several cases of adrenal disease.^7^, ^12^, ^13^ Furthermore, in the present case, AML caused adrenal infarction, which led to adrenal insufficiency. Although the adrenal gland has three inflow vessels, it is anatomically fragile with a single outflow vein and sinusoidal capillaries.^14^ As a result, rapid changes in blood flow can lead to turbulence, which likely predisposes the patient to hemorrhage, infarction, microembolization, and secondary hemorrhage caused by microembolization.^7^, ^14^ Adrenal infarcts are reportedly more likely to occur in pregnant women or patients with antiphospholipid antibody syndrome,^4^, ^15^ and their development is suspected to be related to these patients' procoagulant state.^4^ Although there are few reports of adrenal infarction caused by hematologic diseases such as AML, as in the present case,^2^, ^8^ AML can cause adrenal infarction similarly to other malignancies because of its tendency to induce a procoagulant state.^16^, ^17^ Clinicians' recognition that hematologic disease can cause adrenal infarction and lead to acute abdomen may result in prompt therapeutic intervention.

Our patient had adrenal insufficiency caused by adrenal infarction. The major causes of adrenal insufficiency are autoimmune diseases and infectious diseases such as tuberculosis, cytomegalovirus, and human immunodeficiency virus. Notably, however, adrenal infarction and hemorrhage (which extensively damage the adrenal glands) can also cause adrenal insufficiency, as in this case.^1^, ^18^ Research has shown that 16% to 50% of patients with bilateral adrenal hemorrhage eventually develop potentially life‐threatening adrenal insufficiency.^19^ Similarly, adrenal infarction can also lead to adrenal insufficiency. Even among patients with chronic adrenal insufficiency, 8.3 per 100 patient‐years will develop an adrenal crisis, and 6.3% will have a fatal course^20^; therefore, the chronic course of adrenal insufficiency should be monitored carefully.

Typical symptoms and findings of adrenal insufficiency, including adrenal crisis, are hypotension, shock, weight loss, fatigue, appetite loss, nausea and vomiting, diarrhea, abdominal pain, hypoglycemia, hyponatremia, elevated urea nitrogen, hyperkalemia, and eosinophilia.^1^, ^21^ Our patient had no electrolyte abnormalities, hypoglycemia, or eosinophilia suggestive of adrenal insufficiency. However, the reported frequencies of hyponatremia, hyperkalemia, and hypoglycemia associated with adrenal insufficiency are 72%–88%, 48%–64%, and 62%, respectively, indicating that these abnormalities are not inevitable in all cases.^5^, ^21^ Furthermore, the patient's clinical course was characterized by hypotension, fatigue, and appetite loss, and enlargement of the adrenal glands on CT images suggested adrenal involvement; this led us to start steroid administration on the day of admission. When the adrenal glands undergo ischemia following adrenal infarction, CT, or magnetic resonance imaging often reveals enlarged adrenal glands,^4^, ^5^ hypoenhancing adrenal glands,^11^ mild stranding and a small amount of fluid surrounding the glands,^11^ and a capsular sign represented by a peripheral subtle hyperdense line around a hypodense enlarged adrenal gland^5^ Adrenal infarction can thus be suspected based on these imaging abnormalities. Enlarged adrenal glands are considered to be caused by inflammation and congestion due to occlusion of the adrenal arteries.^5^, ^22^ When enlarged adrenal glands are detected on imaging tests and adrenal insufficiency is clinically suspected at the same time, the patient should be aggressively evaluated for adrenal dysfunction and the cause of the adrenal insufficiency should be identified.

## 4 CONCLUSION

AML can cause acute abdomen following adrenal insufficiency or adrenal infarction. Therefore, when diffusely enlarged adrenal glands and adrenal insufficiency of unknown cause are seen in a patient presenting with acute abdomen, adrenal infarction due to AML, or other hematologic diseases should be ruled out.

## AUTHOR CONTRIBUTIONS

**Risa Hirata:** Conceptualization; writing – original draft. **Masaki Tago:** Conceptualization; project administration; supervision; writing – original draft; writing – review and editing. **Shun Yamashita:** Writing – original draft. **Satsuki Yamamoto:** Writing – original draft. **Shizuka Yaita:** Writing – original draft. **Yuka Hirakawa:** Writing – original draft. **Maiko Ono:** Conceptualization; writing – original draft; writing – review and editing. **Shu‐ichi Yamashita:** Writing – original draft; writing – review and editing.

## FUNDING INFORMATION

There is no funding for this article.

## CONFLICT OF INTEREST STATEMENT

The authors state that they have no conflict of interest.

## ETHICS STATEMENT

This manuscript conforms to the provisions of the Declaration of Helsinki in 1995 (as revised in Brazil 2013).

## CONSENT

Written informed consent was obtained from the patient's wife to publish this report in accordance with the journal's patient consent policy.

## Data Availability

The data that support the findings of this study are available from the corresponding author upon reasonable request.
